# Reciprocal Regulation of Epileptiform Neuronal Oscillations and Electrical Synapses in the Rat Hippocampus

**DOI:** 10.1371/journal.pone.0109149

**Published:** 2014-10-09

**Authors:** Erika R. Kinjo, Guilherme S. V. Higa, Edgard Morya, Angela C. Valle, Alexandre H. Kihara, Luiz R. G. Britto

**Affiliations:** 1 Departamento de Fisiologia e Biofísica, Instituto de Ciências Biomédicas, Universidade de São Paulo, São Paulo, São Paulo, Brazil; 2 Núcleo de Cognição e Sistemas Complexos, Centro de Matemática, Computação e Cognição, Universidade Federal do ABC, São Bernardo do Campo, São Paulo, Brazil; 3 Instituto Internacional de Neurociência de Natal Edmond e Lily Safra, Natal, Rio Grande do Norte, Brazil; 4 Laboratório de Neurociências - LIM 01, Departamento de Patologia, Faculdade de Medicina, Universidade de São Paulo, São Paulo, São Paulo, Brazil; University Paris 6, France

## Abstract

Gap junction (GJ) channels have been recognized as an important mechanism for synchronizing neuronal networks. Herein, we investigated the participation of GJ channels in the pilocarpine-induced status epilepticus (SE) by analyzing electrophysiological activity following the blockade of connexins (Cx)-mediated communication. In addition, we examined the regulation of gene expression, protein levels, phosphorylation profile and distribution of neuronal Cx36, Cx45 and glial Cx43 in the rat hippocampus during the acute and latent periods. Electrophysiological recordings revealed that the GJ blockade anticipates the occurrence of low voltage oscillations and promotes a marked reduction of power in all analyzed frequencies.Cx36 gene expression and protein levels remained stable in acute and latent periods, whereas upregulation of Cx45 gene expression and protein redistribution were detected in the latent period. We also observed upregulation of Cx43 mRNA levels followed by changes in the phosphorylation profile and protein accumulation. Taken together, our results indisputably revealed that GJ communication participates in the epileptiform activity induced by pilocarpine. Moreover, considering that specific Cxs undergo alterations through acute and latent periods, this study indicates that the control of GJ communication may represent a focus in reliable anti-epileptogenic strategies.

## Introduction

Gap junction (GJ) channels couple adjacent cells, allowing transfer of second messengers, ions, and molecules up to 1 kDa. These channels are composed by a multigene family of integral membrane proteins called connexins (Cx). So far, at least 20 Cx genes were identified in the mouse and human genome [1]. Notably, communication through GJ channels has been recognized as an important mechanism for synchronizing neuronal networks in both physiological and pathological conditions [2]–[4]. In fact, several evidences from animal models [5]–[11] and human slices from epileptic patients [12], [13] indicate the participation of GJ channels in the generation and maintenance of epileptic seizures. Moreover, specific alterations of Cx expression have been described in tissue from epileptic patients [14]–[16] and in experimental models [11], [17]–[20].

The aim of this study was to determine the involvement of GJ channels in the epileptiform activity induced by pilocarpine by examining the changes in electrophysiological patterns produced by uncoupling of these channels with carbenoxolone (CBX). After we established the participation of GJ in the ictal discharges, we thoroughly analyzed the regulation of gene expression, changes in protein levels, phosphorylation profile and distribution of the neuronal Cx36 and Cx45 and the glial Cx43, three of the most highly expressed Cxs in the rat hippocampus, [1], [18], [21]–[24] during acute seizures and the epileptogenic process. We observed that pharmacological blockade of GJ channels decreases the epileptiform activity, which in turn regulates Cx gene expression, protein levels and phosphorylation. Thus, our results revealed a reciprocal, mutual regulation of Cx-mediated communication and the epileptiform phenomenon.

## Methods

### Ethics Statement

All experiments were carried out with healthy male Wistar rats (*Rattus norvegicus*) weighing between 270 and 300 g and mean age ranging from 80–90 days. Experiments with animals were conducted in accordance with guidelines of the National Institutes of Health (NIH) and the Brazilian Scientific Society for Laboratory Animals. Experimental protocol (#19, page 67, book 02/2009) was approved by the Ethics Committee in Animal Experimentation of the Institute of Biomedical Sciences/University of São Paulo (ICB/USP). All animals were housed in groups of five in plastic cages under controlled conditions of temperature (22°C±2), relative humidity (45% to 50%) and light/dark cycles (12 hours of light/12 hours of darkness) in a vivarium approved by ICB/USP Ethics Committee in Animal Experimentation. Rats were given *ad libitum* access to a standard rodent maintenance diet (Nuvilab, Curitiba, PR, Brazil) and tap water. Surgeries were performed under diazepam sedation followed by ketamine anesthesia, and all efforts were made to minimize suffering.

#### Implantation of recording electrodes

Animals underwent sedation with diazepam (6 mg/kg, i.p., União Química, Embu-Guaçu, SP, BRA), anesthesia with ketamine (100 mg/kg, i.p., Parke-Davis, Ann Arbor, MI, USA) and stereotaxic surgery for bilateral electrode implantation. All surgeries were performed in the laboratory in the morning. Bipolar 150-µm-diameter nichrome bipolar electrodes were implanted over neocortical area (AP: −1.5 mm, ML: ±3.0 mm) according to skull references [25].

#### ECoG recordings and drug administration

Ten days after the surgical procedure, the rats were placed in a Faraday cage and connected to the input panel of a 21-channel Nihon–Koden electroencephalograph (Neurofax EEG 4400) for habituation 1 day before the experiment. In the day of the experiment, which was always performed in the laboratory between 08 AM and 17 PM, after thirty minutes recording of the basal activity, the first group of animals (SE+CBX; n = 6 animals) was submitted to the injection of methyl-scopolamine (1 mg/kg, s.c.; Sigma-Aldrich, USA), used to reduce the peripheral cholinergic effects, followed by pilocarpine hydrochloride (360 mg/kg, i.p.; Sigma-Aldrich, USA) injection thirty minutes later. Thirty minutes after the establishment of *status epilepticus* (SE), the animals were treated with CBX (60 mg/kg, i.p., dissolved in saline; Sigma-Aldrich, USA) and the ECoGs were continuously recorded for two hours at different time points (0, 30, 60, 90 and 120 min).The second group (SE group; n = 5 animals) was treated in a similar way as the first one, except for the approximate volume of sterile saline (i.p.) injected instead of CBX, and the ECoGs were also continuously registered for two hours. The third group (Control CBX; n = 5 animals) received saline instead of pilocarpine, followed by CBX injection. The behavior of the rats was continuously monitored during the ECoG recordings. Recordings were acquired at 512 Hz, notch filter of 60 Hz, low pass of 35 Hz. A Matlab (Mathworks) script run off line ECoG analysis to detect flat periods longer than 250 ms between the on-SE and off-SE raw recordings for each rat, and minimum time interval for each animal to present flat periods were averaged for both groups. ECoG data were sampled in 5 epochs of 10 seconds to represent each period (basal, methyl-scopolamine, SE, t0, t30, t60, t90, t120) with ECoG power spectrum and spectrogram. Power spectrum analysis was performed using Welch's Method, window 128 and overlap 64. Spectrogram used Kaiser window 1024 and overlap 516. The software to run statistical comparisons as analysis of variance and T-test was SPSS. All statistical comparisons considered significance level set at 5%.

### SE induction for molecular analysis

Animals were previously treated with methyl-scopolamine nitrate (1 mg/kg, s.c.; Sigma-Aldrich, St. Louis, MO) to reduce peripheral cholinergic effects. Thirty minutes later animals of the experimental group received a single dose of pilocarpine hydrochloride (360 mg/kg, i.p.; Sigma-Aldrich, St. Louis, MO) to induce SE, and control animals received a similar volume of sterile saline. SE induction was always performed in the laboratory between 08 AM and 10 PM.

Euthanasia of animals of the acute group was performed by decapitation 2 hours after the onset of SE. Animals of the latent group received a single dose of diazepam (10 mg/kg, s.c., União Química, Embu-Guaçu, SP) 2 hours after SE initiation to interrupt seizures, and euthanasia was conducted 3 days after SE induction.

### RNA isolation, cDNA synthesis and Real-Time PCR

Hippocampi from control (n = 8), acute (n = 8) and latent (n = 8) animals were directly homogenized in 1–1.5 ml TRIzol reagent (Invitrogen, Carlsbad, CA, USA) and total RNA was extracted following the manufacturer suggested protocol and previously described [26]. Briefly, following two chloroform extraction steps, RNA was precipitated with isopropanol and the pellet washed twice in 70% ethanol. After air-drying, RNA was resuspended in DEPC-treated water and the concentration of each sample obtained from *A_260_* measurements. Residual DNA was removed using DNase I (Amersham, Piscataway, NJ, USA) following manufacturer protocol. Quantitative analysis of gene expression was carried out with a Rotor-Gene 6000 Real-Time Rotary Analyzer (Corbett Robotics Inc., San Francisco, CA) with specific primers for rat Cx36 (forward, 5′- GGGGAATGGACCATCTTGGA-3′; reverse, 5′- TCCCCTACAATGGCCACAAT-3′), Cx45 (forward, 5′- GGGTAACGGAGGTTCTGGTTACAGGGTTGTCTACGCCGCC-3′; reverse, 5′- AAGCTCCAACTCATGGTGGT-3′) and Cx43 (forward, 5′-ACTTCAGCCTCCAAGGAGTT-3′; reverse 5′-TGGAGTAGGCTTGGACCTTG-3′). cDNA abundance for cyclophilin A (forward, 5′- GCGTTTTGGGTCCAGGAATGGC-3′; reverse, 5′- TTGCGAGCAGATGGGGTGGG-3′) was determined as internal control. For each 20 µl reverse transcription reaction, 4 µg total RNA was mixed with 1 µl oligodT primer (0.5 µg; Invitrogen) and incubated for 10 min at 65°C. After cooling on ice the solution was mixed with 4 µl 5× first strand buffer, 2 µl of 0.1 M DTT, 1 µl of dATP, dTTP, dCTP and dGTP (each 10 mM), and 1 µl SuperScript III reverse transcriptase (200 U; Invitrogen) and incubated for 60 min at 50°C. Reaction was inactivated by heating at 70°C for 15 min. All PCR assays were performed as follows: after initial activation at 50°C for 2 min and 95°C for 10 min, cycling conditions were 95°C, 10 s and 60°C, 1 min. Dissociation curves of PCR products were obtained by heating samples from 60 to 95°C, in order to evaluate primer specificity. Relative quantification of target gene expression was evaluated using the comparative CT method as previously described in detail [27], [28]. Values were entered into one-way ANOVA followed by Tukey's HSD test, with the significance level set at 5%.

### Western Blotting

Hippocampi from control (n = 7), acute (n = 7) and latent (n = 7) animals were rapidly dissected, washed with phosphate buffered saline (PBS) and homogenized in RIPA buffer (50 mM Tris, 150 mM NaCl, 0.1% SDS, 0.5% sodium deoxycholate, 1% Triton X-100 and protease inhibitors). Homogenates were centrifuged for 20 min at 14,000 G, 4°C to remove insoluble material. Protein concentration was determined by the BCA method (Thermo Scientific, Rockford, IL, USA) and bovine serum albumin was used as the standard, following manufacturer protocol. Proteins in the membrane preparations were separated by sodium dodecyl sulfate-polyacrylamide gel electrophoresis (SDS-PAGE; 10% gel) and transferred to nitrocellulose membranes. Blots were incubated with 5% non-fat milk in TBST buffer for 2 h at room temperature to block nonspecific binding of the antibodies. After rinsed in TBST, blots were incubated overnight with primary antibodies raised against Cx36 (rabbit polyclonal 36-4600, Zymed/Invitrogen, 1∶1000), Cx45 (rabbit polyclonal AB1745, Millipore, Billerica, MA, USA, 1∶1000), Cx43 (rabbit polyclonal 71-0700, mouse monoclonal 13-8300, Zymed/Invitrogen, 1∶1000) and beta-actin (mouse monoclonal A5316, Sigma-Aldrich, 1∶10000) diluted in TBST/3% non-fat milk. After the primary antibody incubation, blots were rinsed in TBST and incubated with the appropriate secondary antibody raised against rabbit or mouse conjugated to horseradish peroxidase (HRP) enzyme (1∶5000, Invitrogen, Carlsbad, CA, USA) for 2 h at room temperature. Detection of labeled proteins was achieved by using the enhanced chemiluminescent system (ECLTM kit; Amersham, Piscataway, NJ, USA). Measurements of optical densities (OD) were performed using Alliance 2.7 system (UVItec Limited, Cambridge, United Kingdom). ODs of the bands were normalized using the value found for the beta-actin. Data from the experiments were entered into T-Test analysis with the significance level set at 5%.

### Immunohistochemistry

Brains were collected and fixed for 8 hours in 1% paraformaldehyde (PFA) in phosphate buffer 0.1 M pH 7.3 (PB), and cryoprotected in 30% sucrose solution for at least 48 hours at 4°C. Brain coronal sections (12 µm) were obtained on a cryostat. Brain sections containing the hippocampus were incubated overnight with primary antibodies raised against Cx36 (36-4600, Zymed/Invitrogen, 1∶100), Cx45 (AB1745, Millipore, Billerica, MA, USA, 1∶200), Cx43 (710700, Zymed/Invitrogen, 1∶100) and parvalbumin (P3088, Sigma-Aldrich, 1∶200) in a solution containing 5% normal goat serum and 0.5% Triton X-100 in PBS at room temperature. After several washes, brain sections were incubated with goat antiserum against rabbit or mouse IgG tagged to Alexa 488 (1∶200–1∶500, Invitrogen) and 3% normal goat serum diluted in 0.3% Triton X-100 in PBS for 2 hours at room temperature. For double-labeling experiments, secondary antibodies conjugated to Alexa 546 or Alexa 647 (1∶200–1∶500, Invitrogen) were used. Controls for the experiments consisted of the omission of primary antibodies; no staining was observed in these cases. Counterstaining of brain sections was achieved using 4′,6-diamidino-2-phenylindole (DAPI), diluted in the same 0.3% Triton X-100 of the secondary antibodies. After washing, the tissue was mounted using Vecta Shield (Vector Labs, Burlingame, CA, USA), and analyzed in Nikon TS100F inverted microscope (Nikon Instruments Inc., Melville, NY, USA). Figures were mounted with Adobe Photoshop CS5 (Adobe Systems Inc., San Jose, CA, USA). Manipulation of the images was restricted to brightness and contrast adjustments of the whole image.

#### Image Quantification

Image analyses were performed with ImageJ software (National Institute of Mental Health, Bethesda, Maryland, USA) and NIS elements (Nikon Instruments Inc.), as previously described [29], employing approximately 30 hippocampal slices from 4 animals (6–8 hippocampal slices from each animal). For Cx36 analyses, after channel separation (RGB) of color images, we performed quantification of the mean pixel intensity, where values correspond to the brightness of the pixels, of Cx36 labeling in the green channel within an area of interest (AOI) defined by the labeling of parvalbumin in the red channel. For analyses of Cx45 and Cx43 images (green channel), we performed quantification of the mean pixel intensity of each AOI (strata oriens, pyramidale, lucidum, radiatum and lacunosum-moleculare). Values were normalized by the mean pixel intensity of the whole image. Mander's colocalization coefficient between Cx36 and parvalbumin was performed. Values from all analyses were entered into one-way analysis of variance (ANOVA), followed by pairwise comparisons in Tukey's HSD test, with the significance level set at 5%. Images and charts were prepared using Adobe Photoshop CS5.

## Results

### GJ communication is involved in the epileptiform activity

As a first approach, we first verified the contribution of GJ communication in epileptiform electrical activity. For this purpose, we induced SE by injecting pilocarpine in two groups of rats, named SE group and SE+CBX group. After pilocarpine administration, animals experienced a progressive evolution of seizures, and approximately 45 minutes later, 80% of the animals had developed SE, characterized by tonic-clonic generalized seizures [30], [31]. Animals treated with CBX without pilocarpine (Control CBX group) did not present behavioral alterations.

**Figure 1:**
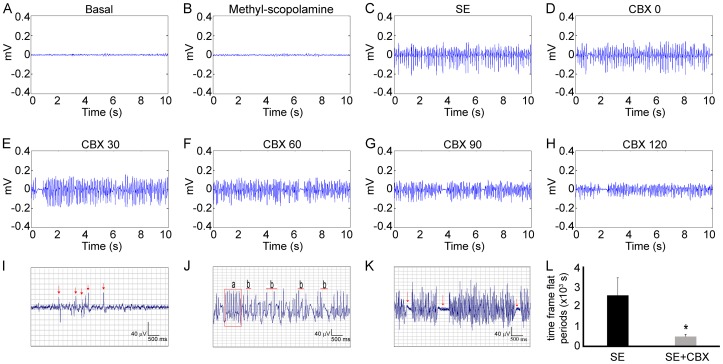
Effects of pharmacological blockade of GJ channels on ECoG. Representative local field potentials of SE+CBX group. (A, B) It is possible to see that basal and methyl-scopolamine periods share similar electrical activity patterns. After pilocarpine injection, we observed increasing epileptiform potentials represented by isolated spikes (I, arrows), poly-spikes (J-a) and spike-wave complexes (J-b), culminating in SE establishment (C). CBX administration 30 minutes after SE establishment (D) was able to induce changes in the epileptiform potentials, which could be seen during the whole analyzed interval post-CBX injection (E–H). We found out that the time frame for occurrence of flat periods (K, arrows) from SE+CBX group was significantly shorter when compared to SE group (L). Bars represent standard errors of mean. *P<0.01 in T-Test.

Figure 1 (A–H) shows the representative ECoGs of SE+CBX group. During the basal and methyl-scopolamine periods, ECoGs display electrical activity of low frequency and amplitude. After pilocarpine administration, ECoGs exhibited increasing epileptiform potentials characterized by isolated spikes (I, arrows) which evolved to continuous poly-spikes and spike-wave complexes (Ja and Jb, respectively), characterizing the establishment of SE (C). Thirty minutes after the establishment of SE, CBX was injected in the animals of SE+CBX group (D). We observed that CBX treatment induced changes in the epileptiform activity, as illustrated during all analyzed periods (E–H). A prominent aspect of the epileptiform potentials also associated with the signal is the occurrence of low voltage oscillations, which resemble flat periods (K, arrows). While we did not detect differences in the total number or duration of these flat periods, the time frame for the manifestation of these events was significantly reduced in the SE+CBX group compared to the SE group, which received saline instead of CBX (476±116 seconds vs. 2565±881 seconds, respectively; *P*<0.01) (L), demonstrating the effects of the GJ blocker on pilocarpine-induced SE. The Control CBX group, which received saline followed by the CBX injection, did not present any of the above described alterations related to CBX treatment (data not shown).

**Figure 2:**
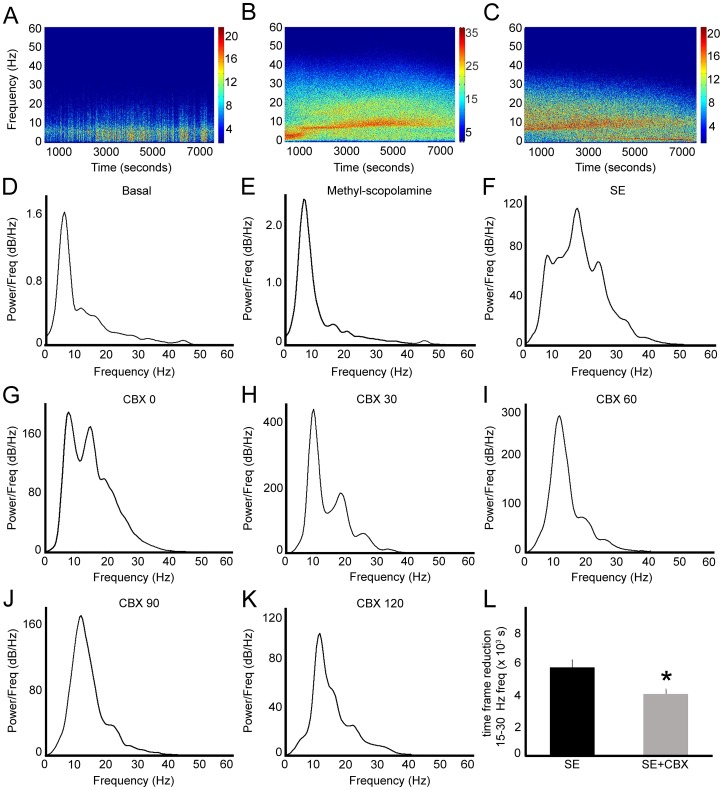
Effects of pharmacological blockade of GJ channels on epileptiform potentials during status epilepticus (SE). (A, B, C) Representative spectrograms of the raw ECoGs from Control CBX, SE and SE+CBX groups, showing the main frequencies, evidenced by the color intensity, which compose the signal during the 2-hour interval after injection of CBX or saline. Note the increase of ECoG power in the SE group (B) and the reduction of color intensity after CBX treatment (C), showing the decrease of power of all frequency bands, especially the beta frequency. Representative power spectra of SE+CBX group. (D, E) Power spectrum of basal and methyl-scopolamine periods shows similar frequency composition. (F) It is possible to observe that pilocarpine-induced SE enhanced the power of all frequency bands, especially at 12–35 Hz range. (G–K) CBX administration 30 minutes after SE establishment caused notable decrease of the power in the beta frequency oscillations observed in the following 2 hours. Since we found that the reduction of beta oscillations occurs during the evolution of SE (data not shown), we tested whether this reduction was significant when compared to the same periods of SE group. (L) We observed that the time period for reduction in the beta frequency range was significantly decreased in the animals that received CBX injection. Bars represent standard errors of mean. *P<0.01 in T-Test.

In addition to the alterations in the epileptiform potentials, we also observed changes in frequency composition. Figure 2 (A–C) shows the frequency composition of ECoGs following 2 hours after CBX or saline injection. (A) The spectrogram of Control CBX group shows greater contribution of low frequencies. On the other hand, the spectrogram of SE group (B) shows high intensity in full range frequencies after saline administration, whereas the spectrogram of SE+CBX group exhibits reduction of intensity of all frequencies that compose the signal (C). In addition, Fig. 2 also shows the representative power spectra of SE+CBX group during all analyzed periods (D–K). We observed that compared with basal and methyl-scopolamine periods (D, E), pilocarpine-induced SE promoted marked changes in the power spectrum (F). Although the potentials observed during the SE period oscillated in the same frequency ranges of the basal and methyl-scopolamine periods (frequencies <5 Hz, 5–11 Hz, 12–35 Hz, known as delta, theta and beta oscillations, respectively), there was a global increase of the power of all frequency ranges. After CBX administration, which occurred 30 minutes after the establishment of SE (G), we visualized changes in the profile of frequency contribution, especially the reduction of the power of frequencies between 15–30 Hz (H–K). Indeed, the time period for the decrease of the power of this frequency band was significantly minor in the SE+CBX group comparing with SE group, which received saline instead of CBX (3925±326 seconds vs. 5599±473 seconds, respectively; *P*<0.01) (L). While CBX administration promoted changes on the above electrophysiological parameters of SE, no behavioral alterations were detected on seizure manifestations.

### Epileptiform activity changes Cxs gene expression during the latent period

**Figure 3:**
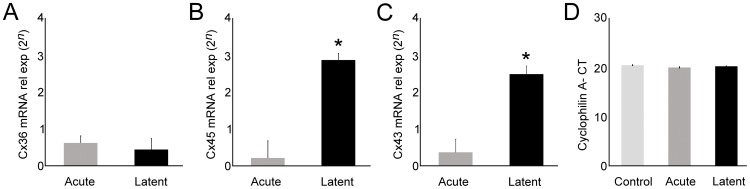
Gene expression levels of Cxs in the rat hippocampus during acute and latent periods. (A) Cx36 gene expression remains stable throughout acute and latent periods. (B) On the other hand, we were not able to detect changes in Cx45 transcript levels in acute period, although increased expression was observed in the latent group. (C) Similar results were observed for Cx43 mRNA levels, since we were not able to detect significant differences in the acute group, but we found increased expression in the latent group when compared to controls. (D) Gene expression of cyclophilin A was used as internal control. Means from acute (n = 8) and latent (n = 8) groups were normalized based on control group (n = 8) expression levels. Bars represent standard errors of mean. *P<0.001 *vs*. Control group in Tukey's HSD pairwise comparisons after one-way ANOVA.

By using primers specifically designed for Cx36, Cx45 and Cx43 we generated amplification plots from cDNA serial dilutions. Dissociation curves of these PCR products were obtained by heating samples from 60 to 95°C. The single peak observed matched to theoretical melting temperature calculated previously, indicating specificity of the primers. Amplification plots indicated that pilocarpine-induced SE did not alter gene expression of any of the analyzed Cxs, as shown in Fig. 3. However, both Cx45 and Cx43 transcript levels have higher expression (2∧2.88 = 7.36 fold-expression and 2∧2.49 = 5.62 fold expression, respectively; P<0.001) in the hippocampus of animals evaluated during the latent period when compared to controls. Cyclophilin A gene expression was used as an internal control (Fig. 3).

### Epileptiform activity induces changes in the total and phosphorylated forms of Cx43

**Figure 4:**
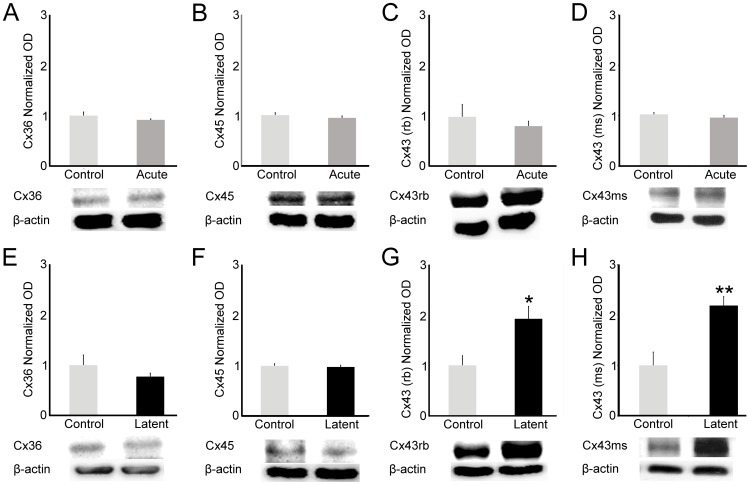
Quantification of Cxs protein levels in the hippocampus during acute and latent periods. (A–D) We observed stable steady levels of Cx36, Cx45, Cx43 protein levels during the acute period. (E–F) Similarly, analysis performed in the latent period showed steady state levels of Cx36 and Cx45 proteins. (G–H) In addition, we observed changes in both Cx43 total protein levels and Cx43 unphosphorylated form, which have higher levels in the latent group when compared to controls. Beta-actin (42 kDa) was used as internal control. Optical densities (OD) from acute (n = 7) and latent (n = 7) groups were normalized by OD from control (n = 7) group in three independent experiments. Bars represent standard errors of mean. * *P*<0.01; **P<0.001 in T-Test.

In order to verify the protein levels of Cxs in the hippocampi of rats during the acute and latent periods, we performed Western blot analysis. We were not able to detect changes in protein levels of the analyzed Cxs in the acute period (Fig. 4, A–D). During the latent period, Cx36 and Cx45 protein levels did not differ compared with control group (Fig. 4, E–G, respectively). However, both Cx43 total protein levels (phosphorylated and unphosphorylated forms, antibody 71-0700) and one of the unphosphorylated forms (antibody 13-8300) of the protein showed higher levels (193%, *P*<0.01 and 168%, *P*<0.001, respectively) when compared to controls (Fig. 4H).

### Cx36 expression in interneurons is not modulated in CA3 by epileptiform activity

**Figure 5:**
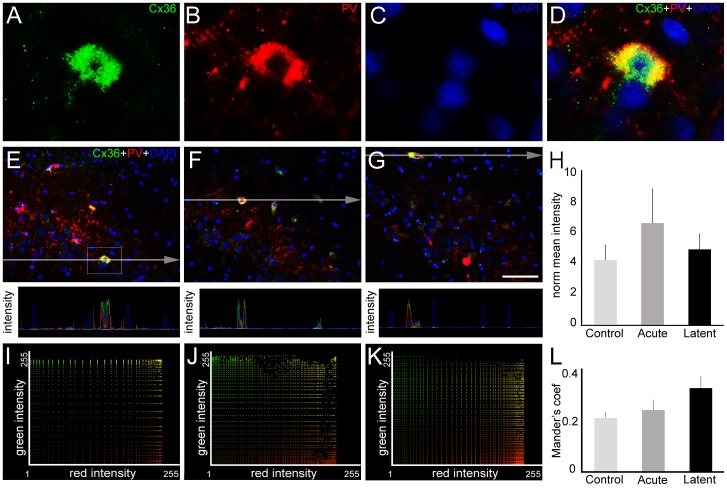
Cx36 expression in parvalbumin (PV)-positive interneurons into CA3. In order to quantify the amount of Cx36 (green) in PV-positive cells (red), we performed double-labeling experiments in coronal sections of rats from control, acute and latent groups counterstained with 4′,6-diamidino-2-phenylindole (DAPI, blue). (A–D) High magnification of the selected area highlighted in the control section (E) shows Cx36 (A), PV (B) and DAPI labeling (C). Colocalization of both proteins is evidenced in (D). (E, F, G) In representative images of CA3 area it is possible to visualize that Cx36 remains in PV-positive cells in acute (F) and latent (G) groups. The pixel intensity profile revealed intersection of the green and red signals, supporting the colocalization of both proteins. (H) Pixel intensity analysis showed no significant differences in Cx36 mean intensity in PV-positive cells between the groups. (I–K) Intensity correlation between green (Cx36) vs. red (PV) channels of representative images from control, acute and latent periods, respectively. (L) Quantitative analysis (n = 4 animals per group) revealed that Mander's colocalization coefficient did not change in acute and latent groups. Bars represent standard errors of mean. Scale bar: 50 µm.

Once we determined that GJ communication is involved in the epileptiform activity induced by pilocarpine and that Cxs undergo alterations in gene expression and posttranslational modifications, we focused on Cx distribution in CA3. For this purpose, we conducted double-labeling experiments using parvalbumin (PV), a calcium binding protein which is known to label a subset of GABAergic interneurons that are electrically coupled by Cx36 [32]–[34]. Indeed, we confirmed the co-expression of Cx36 and PV in the CA3 (Fig. 5, A–D). To determine whether Cx36 amount in PV-positive cells changes in the acute and latent periods, we performed analysis combining pixel intensity profiles and quantification of the mean pixel intensity after RGB decomposition data. We were not able to detect significant differences in pixel quantification analysis (Fig. 5, E–H). Moreover, we did not detect changes in Mander's coefficient from Cx36 and PV signals. Taking together, these results revealed a stable spatial pattern of Cx36 throughout acute and latent periods (Fig. 5, I–L).

### Epileptiform activity induces changes in Cx45 spatial pattern within CA3

**Figure 6:**
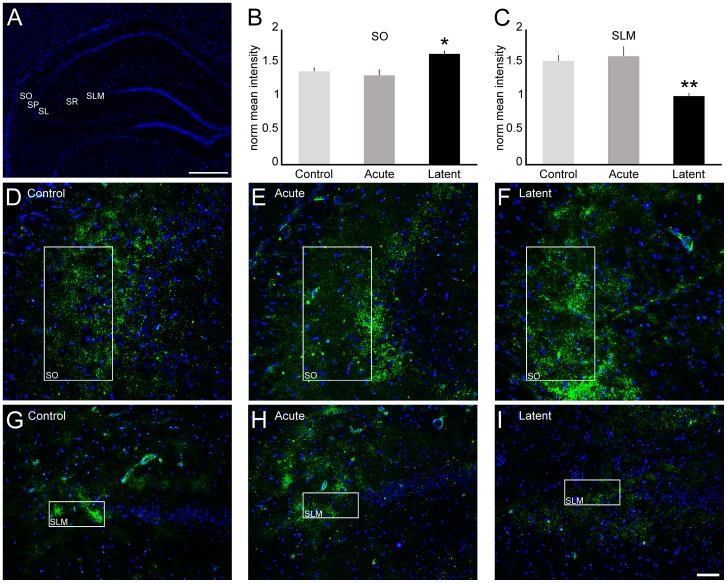
Cx45 distribution in CA3 after epileptiform activity. To examine Cx45 (green) distribution into CA3, we conducted immunofluorescence experiments in coronal sections of rats from control, acute and latent groups counterstained with DAPI (blue). (A) Coronal section labeled with DAPI showing CA3 strata oriens (SO), pyramidale (SP), lucidum (SL), radiatum (SR) and lacunosum-moleculare (SLM). (B, C) Quantification of mean pixel intensity (n = 4 animals per group) showed significant increase in (F) SO and decrease in (I) SLM in the latent period, compared with control animals (D, G). In these subfields, we were not able to detect differences in the acute period (E, H), as well as in the other strata in both acute and latent periods (data not shown). Bars represent standard errors of the mean. *P<0.05, ** *P*<0.01 *vs*. Control group in Tukey's HSD pairwise comparisons after one-way ANOVA. Scale bar: 50 µm.

As we observed widespread Cx45 immunoreactivity in CA3 region, we conducted quantification of the mean pixel intensity in each strata (Fig. 6A) to examine whether Cx45 distribution is altered in the acute and latent periods. We were not able to detect changes in Cx45 distribution during the acute period (Fig. 6B, 6C and data not shown). During the latent period, our quantitative analyses revealed more pronounced labeling in the stratum oriens (119%, *P*<0.05), as well as decreased staining in stratum lacunosum-moleculare (65%, *P*<0.01) when compared to controls (Fig.6, B–I).

### Epileptiform activity induces changes in Cx43 spatial pattern within CA3

**Figure 7:**
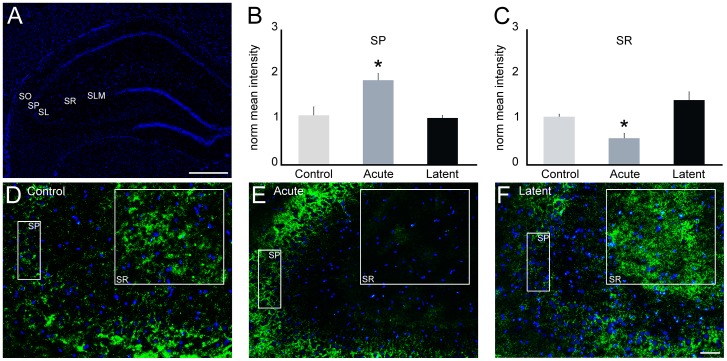
Cx43 distribution in CA3 after epileptiform activity. Immunofluorescence experiments were performed to analyze the distribution of Cx43 (green) in coronal sections of rats from control, acute and latent groups counterstained with DAPI (blue). (A) Coronal section labeled with DAPI showing CA3 strata oriens (SO), pyramidale (SP), lucidum (SL), radiatum (SR) and lacunosum-moleculare (SLM). (B, C) Quantification of mean pixel intensity (n = 4 animals per group) showed significant increase in SP and decrease in SR in the acute period (E) compared to controls (D). In these subfields, we were not able to detect changes in the latent period (F), as well as in the other strata in both acute and latent periods (data not shown). Bars represent standard errors of mean. *P<0.05 *vs*. Control group in Tukey's HSD pairwise comparisons after one-way ANOVA. Scale bar: 50 µm.

To further examine the regulation of specific Cxs within the CA3 region, we evaluated the distribution of Cx43 in CA3 subfields, similarly to the analysis performed for Cx45. The quantification of the mean pixel intensity in each strata (Fig. 7A) revealed that during the acute period, Cx43 immunolabeling was increased in stratum pyramidale (171%, *P*<0.05), whereas reduction of 48% (*P*<0.05) was observed in stratum radiatum (Fig. 7, B–E). In the latent period, we were not able to detect significant changes in Cx43 distribution (Fig. 7, B–D, 7F and data not shown).

## Discussion

The main purposes of this work were to demonstrate the involvement of GJ communication in the epileptiform activity and the possible alterations in Cx gene expression, protein levels, phosphorylation profile and distribution in the hippocampus triggered by pilocarpine. Indeed, our results showed that blockade of Cx channels produced antiepileptiform effects, indisputably correlating the GJ coupling with the epileptiform activity induced by pilocarpine. In spite of previous evidences in the literature regarding the anticonvulsant effects of GJ blockers, based on both in vitro [5]–[9], [35] and in vivo models [10], [11], this is the first report addressing this issue in the pilocarpine model, which reproduces most of the characteristics of human temporal lobe epilepsy [30], [31], [36], such as histopathological features associated with neuronal network reorganization, the occurrence of an initial precipitant injury (the acute phase), a seizure-free interval (latent period), and the occurrence of spontaneous recurrent seizures (chronic phase).

The mechanisms governing oscillations at the cellular and network levels underlie processes related to cognitive and pathological brain states [37], [38]. In this study, we were able to detect a marked increase in the power of beta frequency band following pilocarpine administration. Accordingly, a pronounced contribution of 20–80 Hz frequency band was reported in the kainic acid and in the lithium-pilocarpine models of temporal lobe epilepsy [39], [40]. Furthermore, we also showed that GJ communication participates in this oscillatory activity, since blockade of GJ channels promoted a significant decrease in the power of several frequencies, especially in the beta range.

Another evidence supporting the involvement of GJs in the epileptiform activity presented herein was the anticipation of the low voltage periods after CBX treatment. These events resemble the flat periods previously described [41]. According to that study, flat periods occur in the last two of the five electrographic stages described, namely continuous ictal discharges with flat periods and periodic epileptiform discharges on a flat background, which could be related to the increased inhibitory activity due to the prolonged SE.

Several studies deliberate about the optimal electrographic pattern after management of refractory SE with standard clinical treatments. A number of works point the EEG burst suppression pattern as the model for electrographic pattern (suppression consists of a flat EEG pattern – [42]–[46]), although others support a flat record pattern, which comprises total suppression of EEG signal background [47]. In spite of these controversies, our results regarding anticipation of flat-like periods after GJ blockade exhibit a certain similarity with the EEG recording expected after SE standard treatment, suggesting that Cx channels could serve as a potential target in refractory SE management.

Moreover, the earlier onset of flat periods after CBX treatment observed in our study could be explained by a hyperpolarization over a large number of cortical neurons [48]. It is known that GABAergic interneurons are important players in the regulation of cortical principal cells discharge. Additionally, synchronized activity of GABAergic interneurons is a mandatory feature that leads to synchronization of action potential of principal cells [49]–[51]. GJ composed of Cx36 are important structures that participate in the regulation of GABAergic interneuron synchronization [32]–[34]. In fact, disruption of GJ communication by deletion of Cx36 promotes a higher inhibitory response in neurons during high frequency discharges [52], [53]. Therefore, our results obtained with CBX treatment might be due to the desynchronization of GABAergic interneurons following uncoupling, demonstrating an important role of GJ communication in epileptiform potentials.

Although it has been demonstrated that CBX has effects other than on GJ channels, such as on intrinsic neuronal properties [54], antagonism of GABA_A_ receptors [20], blocking of calcium channels [55] and on endogenous glucocorticoid metabolism [56], there are also evidences that CBX does not affect neuronal excitability and GABA responses [6], [57], [58], and that the depressed spontaneous epileptiform activity observed in hippocampal slices was not due to the mineralocorticoid agonist action of CBX [59]. In spite of these controversies, CBX has been widely used in GJ studies, where neuroprotective effects were examined [60], often in parallel to the demonstration of its uncoupling properties [35], [61], [62]. Indeed, the effects of CBX and its analogous on neuronal coupling and synchronization convincingly recapitulated Cx KO models [62], [63]. Taken together, these independent evidences from distinct groups allows for the use of CBX in GJ studies [64], [65].

In addition to the participation of GJ communication in the epileptiform activity, we also determined several changes in Cx expression during the acute and latent periods. Although there are studies pointing to the participation of Cx36 in epileptiform discharges [19], [66]–[68], we were not able to detect changes in Cx36 mRNA and protein levels. Furthermore, the spatial pattern distribution of Cx36 throughout acute and latent periods remains constant, evidencing the stability of this protein in conditions of epileptiform discharges and subsequent epileptogenesis. However, considering that pilocarpine model induces neuronal loss [69]–[73], Cx36 stability might reflect an important role of GJ communication in the networks of GABAergic interneurons and principal cells that express this protein. Moreover, it is possible that even small differences in Cx expression play a significant role in the network coupled by electrical synapses [17], which in turn could participate in the seizure activity and the following process of epileptogenesis.

In agreement with previous studies, we detected the presence of Cx45 in hippocampal neurons [18], [23], [74]. Moreover, we detected changes in Cx45 distribution in the SO in the latent phase, which is consistent with the enhanced Cx45 transcript levels. Indeed, electronic coupling via GJ was reported between SO interneurons presumably in dendrites [75]. Additionally, the typical low pass filter feature imposed by GJ coupling over of the signal conductance could support synchronization of slow oscillations in the distal dendrites between SO interneurons. Also, it was reported the possible involvement of GABAergic interneurons presumably coupled by GJ in the slow oscillations recorded in hippocampal pyramidal cells [76]. Thus, our data regarding increase of Cx45 in SO could indicate the enhancement of coupling between the interneurons, which might intensify the occurrence of slow oscillations that, in turn, are noticed in a variety of epileptic activities [77], [78].

Furthermore, the expression of Cx45 in the SO hippocampal region, as pointed out in our work, could represent a substrate for the GJ coupling between axons of principle cells previously reported in the hippocampus [57], [79]. During the latent period, we observed an increased amount of Cx45 in this region. Interestingly, collateral connections from CA3 to CA1 are located within SO, and this is probably the site of occurrence of GJ connections [57]. Taking together, if the axo-axonal coupling that possibly takes place at SO involves Cx45, the upregulation of this protein could account for the generation of high frequency oscillations and the increased excitability observed in epileptic conditions [79].

Contrasting the increased levels in the SO, we noticed a decrease of Cx45 in the SLM during the latent period. It is well established that interneuron-mediated GABAergic synchronous potentials might play an important role in epilepsy [80], [81], and that the mechanisms underlying these responses could be mediated by electrical coupling [82], [83]. Indeed, propagation of GABAergic synchronized potentials recorded from SLM seems to be under control of electrical coupling [83], [84]. Interestingly, the synchronous synaptic release of GABA from interneurons of SLM promoted CA3 pyramidal cell activation [84]. Thus, the reduction of Cx45 could lead to desynchronization of GABA release from the interneurons, consequently decreasing CA3 pyramidal cell activity. Therefore, downregulation of Cx45 in the SLM during the latent period could indicate a plastic homeostatic change of the coupled network in order to restore its activity after the epileptogenic insult, since interneuron networks within SLM could regulate the input from the entorhinal cortex to the hippocampus [85].

Whether modulation of Cx45 expression in SO and SLM remains at later time points (e.g. from the time that animals present spontaneous recurrent seizures) is not known, but it would be worth evaluating the issue.

Interestingly, whereas changes in Cx45 distribution were seen in the latent period, alterations in Cx43 distribution through CA3 layers were observed in the acute period. Astroglial networks provide the supply of glucose and lactate necessary for the maintenance of hippocampal synaptic activity in an activity-dependent manner, and it has been shown that this metabolic network of astrocytes is mediated by GJ composed by Cx43 and Cx30 [86]. Thus, the increase in Cx43 distribution pattern observed in the SP is probably due to the high energetic demand promoted by the sustained epileptic activity. Additionally, the coupled astrocytes could provide an important intercellular pathway for both delivery of metabolites to distant locations during episodes of epileptic discharges and buffering of ions such as potassium [87], [88]. Increase of Cx43 in SP could account for two opposite situations: (1) besides providing distribution of metabolites of neurotransmitters and potassium (for review see ref. [89], [90]) to distant locations during episodes of epileptic discharges, a Cx43 increase could lead to the formation of GJ hemichannels, a pathway for adenosine release [91], which could also contribute to the local neuroprotective effects; (2) by allowing the propagation of toxic metabolites and death signals to adjacent cells, the increase in astroglial network coupling could amplify the damage to more distant sites, in addition to the possible contribution to the rapid propagation of the electrical signal [15].

Contrary to the observed in SP, we found decreased labeling of Cx43 in the CA3 SR. It has been demonstrated that the contribution of GJ in potassium buffering in the hippocampus is layer-dependent [92]. By comparing the properties of astrocytes from mice with Cx30/Cx43 deficiencies, those authors found that single astrocytes from SR reach larger areas than those from SLM, indicating the unequal size and orientation of astrocytes from those areas. Additionally, they found no impairment of potassium buffering in SR of transgenic mice, in contrast to the observed in the SLM, evidencing the participation of other elements in the potassium redistribution in the SR. Therefore, the reduction of Cx43 labeling noticed here in the SR is unlikely to have a great impact in spatial buffering of potassium during SE; moreover, as CA3 SR is well supplied by a vascular source [93], which in turn accounts for the degree of astrocyte coupling [94], such decrease of Cx43 labeling in this layer during epileptic activity may not have a significant impact in the physiology of CA3 SR cells.

Remarkably, our results disclosed enhancement of the non-phosphorylated form of Cx43 during the latent period, suggesting reduction of astrocyte coupling, since the unphosphorylated form is not related with the assembly into GJ [95], [96]. This result is quite in accordance with the immunofluorescence data, once both point to a smaller participation of Cx43-mediated GJ during the latent period, despite the increase in transcript levels at the same period, which could account for upregulation of protein levels in later time points.

In summary, we demonstrated the involvement of GJ channels in a model of temporal lobe epilepsy by describing the antiepileptiform effects of GJ blockade on pilocarpine-induced SE. Additionally, we depicted alterations of specific Cxs genes and protein, and changes in Cx distribution at different sites within the hippocampus throughout acute and latent periods. Taken together, our results revealed a reciprocal regulation of neuronal oscillations and electrical synapses, indicating that the control of Cx-mediated communication may take part in reliable anti-epileptogenic therapies.
